# Interpretable multi-horizon time series forecasting of cryptocurrencies by leverage temporal fusion transformer

**DOI:** 10.1016/j.heliyon.2024.e40142

**Published:** 2024-11-05

**Authors:** Arslan Farooq, M. Irfan Uddin, Muhammad Adnan, Ala Abdulsalam Alarood, Eesa Alsolami, Safa Habibullah

**Affiliations:** a Institute of Computing, Kohat University of Science and Technology, Kohat, 26000, KP, Pakistan; b College of Computer Science and Engineering, University of Jeddah, Jeddah, 21959, Saudi Arabia; c Department of Information Systems and Technology, College of Computer Science and Engineering, University of Jeddah,Jeddah, 21959, Saudi Arabia

**Keywords:** Block chain, Deep learning, Cryptocurrency, Sentiment analysis, Artificial intelligence, Transformers, Temporal data

## Abstract

This research delves into the obstacles and difficulties associated with predicting cryptocurrency movements in the volatile global financial market. This study develops and evaluates an advanced Deep Learning-Enhanced Temporal Fusion Transformer (ADE-TFT) model to estimate Bitcoin values more accurately. This research employs cutting-edge artificial intelligence (AI) and machine learning (ML) techniques to comprehensively examine various aspects of cryptocurrency forecasting, including geopolitical implications, market sentiment analysis, and pattern detection in transactional datasets. The study demonstrates that the ADE-TFT model outperforms its lower-layer counterparts in terms of forecasting accuracy, with reduced Mean Absolute Percentage Error (MAPE), Mean Squared Error (MSE), and Root Mean Square Error (RMSE) values, particularly when using a higher hidden layer configuration (h=8). The study emphasizes the importance of experimenting with different normalization strategies and utilizing various market-related data to enhance the model's performance. The results suggest that improving forecasting accuracy may require addressing these limitations and incorporating additional factors, such as market sentiment. By providing investors with more precise market predictions, the techniques and information presented in this research have the potential to significantly increase investor power in an unpredictable digital currency market, enabling wise investment choices.

## 1 Introduction

Investors worldwide experience significant financial uncertainty because of the drop in traditional currency values and vulnerabilities in the stock market. This unstable environment has sparked the transition to digital currency as a novel alternative. The world saw the future of decentralized financial transactions with the launch of Bitcoin (BTC) in 2009 [43]. Blockchain technology, which is the foundation of cryptocurrencies, introduces two key changes from conventional financial systems [47]. First, they are entirely digital, allowing for instantaneous global transactions that get around the restrictions and delays associated with traditional banking. Every transaction is recorded on a public ledger maintained by a decentralized computer network. Second, transactional security offered by digital currencies is unmatched because of their inherently cryptographic nature.

Any deception or fraud is extremely difficult because it requires validation from the entire network. Digital currencies also avoid the difficulties associated with conventional finance, such as changing exchange values and interest rates. Many even provide the extra benefit of low or no transaction costs. After Bitcoin's launch, the world of cryptocurrencies exploded, with a wide variety of other virtual currencies. This progression sparked an increase in computer science research. Forecasting has several facets in the world of cryptocurrencies. It involves more than just forecasting price changes based on past data; it also considers studying market sentiment, comprehending geopolitical developments that affect the adoption of digital currencies, and spotting patterns in sizable transactional datasets. Forecasting is a dynamic and ever-evolving field, and advanced ML and AI techniques are being used to increase prediction accuracy. However, Bitcoin's notorious volatility has deterred substantial institutional investors, resulting in a market landscape dominated by less-experienced traders [2]. Such a shift has ramifications for the trading volume and overall market stability. Against this backdrop, this study developed a robust forecasting model for the cryptocurrency market. We aim to empower investors to anticipate pivotal market events and make judicious investment decisions based on informed forecasts.

With the continuous expansion of deep learning (DL), various relevant learning methods, such as Long Short-Term Memory (LSTM), Temporal Convolutional Networks (TCN), and transformers, have been enhanced and deployed in a manner that is more appropriate for extracting time-series information. LSTM is an improved RNN that can be trained to extract relevant information from the temporal data automatically. It can be used to analyse time series and works by accepting inputs related to past and future states, thus considering attributes related to time [21]. TCN are networks that focus on interpreting sequence data and provide high parallel computing capabilities, a large receptive field, and a constant gradient that LSTM lacks [35], [24], [20]. In addition, some researchers have begun to use attention mechanisms for sequence problems because they perform well in computer vision and natural language processing [46], [27]. The Transformer model improves the interpretability of the model by classifying a significant portion of the input for each instance, depending on the size of the attention weight. In terms of capturing long-distance dependencies, the transformer model performed noticeably better than the RNN approach.

Due to several features such as lack of trustworthy indications, high unpredictability, and multiple affecting elements including technology improvements, market pressures, use patterns, expenses, security concerns, and cultural circumstances, price prediction for cryptocurrencies is a difficult endeavour. Cryptocurrencies have more unpredictability than conventional financial projections, such as stock market forecasts, making investments riskier and less rewarding [3]. Owing to their inherent volatility and scarcity of historical data, cryptocurrency values cannot be predicted with any degree of accuracy.

The proposed work's main objectives are:

1. To determine the precision of the ADE-TFT model, future results will be forecast.
2. To demonstrate the importance of variables in forecasting using the interpretation of the model.
3. To evaluate the model performance using supervised metrics techniques.

## 2 Background and related work

### 2.1 Multivariate time series

Time-series data can be univariate or multivariate. Single data points can be seen in the first scenario, but many data points are acquired for each iteration of the observation process in the second [5]. Considering the important factors for prediction, it is likely that the results will improve. One may argue, for instance, that how much salmon is purchased at a supermarket during a given day depends not only on how many replacements are purchased at that time but also on how many customers are there. The financial time series' final stock price may also be influenced by the stock's open, high, low, and volume and its historical value. In multivariate time-series systems, spatial and temporal features can be utilized for more information to improve forecasting. Temporal connections illustrate how well the variables vary over time, whereas spatial relations demonstrate how one variable affects others. Each time step in multidimensional data is expressed as a vector, $Z_{t}=(z_{1t},z_{2t},...,z_{kt})$, where k denotes the total number of features used in the prediction [8]. Consequently, the characteristics and implementation of each series were considered. This suggests that the lag among the time series should be used to determine the covariance between them rather than the time interval between them [40].

### 2.2 Financial time series

In time-series literature, price movements are a typical illustration of non-stationarity and nonlinearity. Additionally, they are notable for having a lot of noise [8], [13], [29]. Financial time series contain impacts and causes that can be identified by data analysis. For instance, [9] discovered daily trading trends between volume trading and returns for stock market indices of foreign currency, and [18] discovered a lead-lag correlation between both Bitcoin and commodity markets. Many investors use chart patterns to determine market-shifting moments and their objectives. It is possible to analyze the differences between a stock's short- and long-term moves using multiple moving average windows. Additionally, the use of Bollinger Bands, an indicator that captures the price and volatility history of a financial asset, is widespread [15], [23].

### 2.3 Traditional forecasting methods

Traditional forecasting techniques can derive linear patterns from data using historical processes. The single-line formula, which is adapted to the data's simplicity of understanding, is a benefit of conventional methods. They are also user-friendly and memory-efficient.

### 2.4 Autoregression and moving average

A statistical technique called autoregression is used to forecast a variable's future value based on its past values while analyzing the time series. This is a linear regression model; in other words, it employs lag variables as predictors. The word “autoregression” derives from the fact that the variable's current value is based on its prior values, hence the “auto” in the phrase. An order p autoregressive model, designated by AR(p), can be expressed mathematically as

(1) $$y_{t}=b+\phi_{1}y_{\left(t-1\right)}+\phi_{2}y_{\left(t-2\right)}+...+\phi_{p}y_{\left(t-p\right)}+\epsilon_{t}$$

where $y_{t}$ is the relevant variable at time t, b is a positive constant, $\phi_{1},\phi_{2},...,\phi_{p}$ are the vectors of autoregressive coefficients, $\epsilon_{t}$ is the error term, and p is the order of the autoregression. The error term $\epsilon_{t}$ is assumed to be white noise, which means it has a mean of 0 and constant variance and is uncorrelated with the lagged values of y. The autoregressive coefficients $\phi_{1},\phi_{2},...,\phi_{p}$ represent the impact of the past values of y on the current value. A positive autoregressive coefficient for a particular lag indicates that the current value of y is positively related to the value at that lag. Conversely, a negative coefficient indicates a negative relationship. The time series data autocorrelation function (ACF) and partial autocorrelation function (PACF) were used to determine the order of autoregression. While the PACF calculates the correlation between a variable at a specific lag and its previous lags, the ACF calculates the correlation between variables at various lags. We can determine the correct order of autoregression by analyzing the ACF and PACF plots.

In contrast to the autoregressive (AR) model, the Moving Average (MA) takes the average of a specified number of prices over a given time. The result is a line that tracks the average price of an asset over a specified time, which can help identify trends and provide signals for trading. Specifically, an MA model of order q is expressed as a linear combination of the q previous forecasting errors.

(2) $$y_{t}=\mu +\theta_{t}\epsilon_{t-1}+\theta_{t}\epsilon_{t-2}+...+\theta_{t}\epsilon_{t-q}+\epsilon_{t}$$

In the given equation, the parameter $\theta_{t}\in R$ represents the model parameters, while $\epsilon_{t}∼N(0,\sigma^{2})$ [1], [22] is the predictive error that has a mean of 0 and a variance of $\sigma^{2}$ under a standard deviation. White noise is used to model these errors. Additionally, *μ* stands for the time series' average value.

Autoregressive (AR) and Moving Average (MA) models are frequently combined to produce complex models. These models include the ARMA model [22], ARIMA model [42], and ARIMAX model [44]. The ARMA model, which combines the MA(q) and AR(p) models, is the most fundamental among the three models.

(3) $$\mathrm{ARMA}(p,q)=\mathrm{AR}(p)+\mathrm{MA}(q)=c+\epsilon_{t}\sum_{i=0}^{p}\phi_{i}y_{t-i}+\sum_{j=1}^{q}\theta_{t}\epsilon_{t-j}$$

The Exponential Smoothing (ES) approach is an alternative to the MA (Moving Average) and autoregressive (AR) models. In ES, the weights assigned to past observations are not uniformly weighted, as in the AR and MA models. Instead, the weights in the ES models were exponentially reduced over time. The Simple Exponential Smoothing (SES) model [22], [26] is one of the simplest ES models, and is suitable for time series that do not exhibit a trend. However, more complex models such as the Double Exponential Smoothing (DES) model are more appropriate for time series with a trend.

(4) $${\overset{ˆ}{y}}_{t}+1=\alpha y_{t}+(1-\alpha )y_{t-1}+{\left(1-\alpha \right)}^{2}y_{t-2}$$

Reddy et al. compared the LASSO machine-learning algorithm with other models, such as QUANDL, RNN, SVM, and CNN. LASSO offers excellent time management, allowing it to get superior results from a large dataset. They have the highest level of accuracy in predicting daily trending changes in the Bitcoin market [39]. Derbentsev forecasted the bitcoin values by using two machine learning algorithms, stochastic gradient boosting machine (SGBM) and random forest (RF). The results demonstrate that machine-learning approaches can estimate Bitcoin values. However, the decisions were made at the appropriate time to reduce the risks associated with the investment decision [14].

Kumar et al. demonstrated that everyday data comprised 1000 data samples, hourly data of 1500 data samples, and minute data of 400000 data lines. They discovered how DL algorithms enabled pricing trends in the Ethereum cryptocurrencies. For forecasting Ethereum value and mapping Mean Absolute Percentage (MAPE) errors, they used Multi-Layer Perceptron (MLP) or Long Short-Term Memory (LSTM) models [25].

A study conducted by Ahmed B. et al. performed a comparative analysis of deep learning and ensemble learning models for predicting various cryptocurrencies [6]. The results showed that ensemble learning and deep learning models perform better than shallow neural networks and traditional statistical methods. If there is less complexity in the time series cryptocurrency data then conventional statistical methods can achieve better results for regression metrics. The study concluded that LightGBM outperformed Bitcoin, Litecoin and Ethereum cryptocurrencies while GRU showed the best result for Ripple.

Another study by Lucas D. A. Takara et al. argued that advanced computation models based on deep reinforcement learning (DRL) are better in decision-making, particularly in quantitative trading [12]. If properly trained on past data, DRL can automatically develop profitable strategies for trading. The authors presented a customized and innovative model called Extended Trading DQN (ETDQN), based on the Deep Q-Network (DQN) algorithm. The particular feature of ETDQN was that it was dynamic and adapted to changing market conditions. Moreover, ETDQN receives feedback only when trades are completed, enabling the model to perform better trade decisions. The distributed learning feature of ETDQN enables it to improve decision-making and only focus on maximizing profits.

Other studies such as [16] and [41], [17] used various ML models such as GARCH, HAR, SVR, LASSO, MLP, RF, and LSTM to forecast cryptocurrency volatility that will help investors in making the right and informed investment decisions. Because there are several cryptocurrencies, no single ML model is best for all cryptocurrencies. The studies revealed that different models perform differently depending on forecast context and error metrics. Interestingly, simple models such as linear regression and ridge regression show similar results to that of complex ML models such as LSTM and RF.

Lopez and other scholars collected data from six different facilities in Germany and Australia by applying the TFT model to predict hourly day-ahead PV power generation with statistical error indicators to compare the outcomes with other models. TFT has shown more precise results than the other algorithms to forecast PV power generation [31].

Furthermore, Feng addressed the supply air temperature in high-speed train carriages by enhancing two TFT architecture components: the Double-Convolutional Residual Encoder and the spatio-temporal double-gate. Additionally, a loss function is also developed which is appropriate for generic long-sequence time-series forecast tasks for predicting temperature, that increases MAPE by 11.73% and 21.70% compared to the original model [19].

[45] suggested a novel wind speed prediction system that uses VMD, adaptive DE (ADE), and TFT algorithms. The system distinguishes the significance of each meteorological variable for wind-speed forecasting by considering past and present wind-speed data and a variety of climate parameters simultaneously. While ADE optimizes the TFT model variables, VMD divides the raw wind speed information into various band-limited mode functions. The proposed approach performs more effectively than other similar models, demonstrating its consistency and dependability. Additionally, it identifies essential aspects in predicting wind speed, supporting decision-makers in their choices.

[49] proposed transformer-based forecasting models for cryptocurrencies. The model was fed with historical tweet data as well as cryptocurrency price information. The proposed model makes predictions using attention mechanisms to identify significant patterns and features in the input data. The model was tested using two different datasets, Ethereum and Bitcoin, with encouraging outcomes. The accuracy of the model, which outperformed other approaches, was 75.87% for Bitcoin and 78.55% for Ethereum. According to the study, adding sentiment analysis to the prediction model can significantly increase the accuracy of predicting cryptocurrency prices.

[20] proposed two deep models: the LSTM network, which was used with deep multi-input, multi-output (MIMO), and multi-input single-output (MISO) architectures, and the TCN network, which was combined with the MIMO and MISO architectures for long-term forecasting. These models, which can be run on a standalone computer, accept up to 10 parameters as input and output a single parameter as a prediction. The data source was a set of daily weather records from South Korea from 2011 to 2018. The proposed models produce 9-hour weather forecasts that are trustworthy and accurate predictions, which have not previously been investigated in weather forecasting.

[30] studies propose deep learning models like LSTM, BiLSTM, and convolutional layers in an ensemble learning approach to forecast cryptocurrency prices. Ensemble models are assessed on both classification and regression tasks and are based on three widely used ensemble learning strategies: ensemble averaging, bagging, and stacking. The authors agree that careful feature engineering and hyperparameter tuning are necessary to enhance the prediction accuracy of the ensemble models. Choosing the quantity and type of base learners in an ensemble strategy is important for precise and trustworthy predictions, but it can also impact the cost and speed of computation. Owing to its accuracy and dependability, this approach holds promise for low-frequency applications, even though it might not be feasible for high-frequency real-time applications.

[34] uses semantic analysis and machine learning approaches to investigate the consistency of Bitcoin's USD price direction. This study concentrates on the Bitcoin closing price and sentiments of the present market to create a forecasting model. Twitter and Reddit were used to collect data on popular opinions on Bitcoin. The findings demonstrate that in comparison to the ARIMA model's RMSE of 209.263, the LSTM with multiple features produces a more accurate prediction, with an RMSE of 197.515. The report admits that the model's accuracy and consistency would be enhanced by the capacity to anticipate data streaming. The author also acknowledges that it may be biased to only look at tweets and posts on Reddit and Twitter and that adding data from LinkedIn and Facebook posts might yield a more complete picture of public opinion. Finally, the study found that LSTM is a more effective and accurate model than ARIMA for predicting Bitcoin's USD price direction.

[33] attempted to anticipate the daily movements in Bitcoin, Ethereum, and Ripple prices using LSTM networks using various factors, including sentiment, power prices, financial instability, and historical price data. In addition to sentiment data that went beyond the individual cryptocurrencies being forecasted, the article was distinguished for its usage of an original variable set that had never been examined previously. The findings show that the top-performing models for Ethereum and Ripple had accuracy levels of over 50% overall and up to 72% in forecasting rising Ethereum trends, which might help cryptocurrency investors make better decisions. However, these models were not sufficiently precise for automatic trading. The generated models showed overfitting symptoms, proving that the particular collection of variables combined with LSTM was unreliable for forecasting Bitcoin trends. Ultimately, by demonstrating the potential of LSTM networks and our weeksits in foretelling Bitcoin trends, this study made a significant addition to the area of cryptocurrency prediction.

[10] authors' use of a multivariate method for estimating patient volume in public hospitals by leveraging the deep neural network architecture of the Temporal Fusion Transformer, on the dataset of Emergency Departments (EDs) of Portuguese public medical facilities by Health Regional Areas (HRA). For four weeks, the model projected forecast intervals and precise predictions using covariates from the calendar and time series. With a Mean Absolute Percentage Error (MAPE) of 5.90% and a Root Mean Squared Error (RMSE) of 84.4102 individuals/day, the results demonstrated that the model outperformed other frequently encountered models discussed in the literature. By including more pertinent static variables and enlarging the forecast window, this study also demonstrates the possibility of future advancements.

[32] examined the predictability of financial returns for six significant digital currencies Binance Coin, Bitcoin, Cardano, Dogecoin, Ethereum, and Ripple during the pre-COVID-19 and ongoing COVID-19 periods. In contrast to conventional forecasting, this study suggests a novel method to identify cryptocurrency returns that fall within the first, second, third, or other quantiles of gold prices the following day. The support vector machine (SVM) technique was used to analyze the data, and the proposed algorithm enabled updated data analysis by utilizing sensors in the database. The study results confirm that the SVM algorithm is a reliable method for building profitable trading approaches, and can be applied with the help of algorithms to achieve a reliable estimate before or during a global pandemic [7]. During challenging times such as the COVID-19 crisis, stakeholders who are keen on following Bitcoin trends may benefit from this research by enhancing their understanding of Bitcoin dynamics and subsequently making wise investment decisions.

Another related work conducted by [28] examined the prediction of Bitcoin price changes due to significant fluctuations using fundamental market indicators as well as technical features obtained through a denoising autoencoder. The Attentive LSTM network and Embedding Network were used by the authors to evaluate the features. (ALEN). ALEN surpasses all baselines by gathering hidden representations from related currencies and capturing the representation of Bitcoin. This study explores the effects of several characteristics on the forecast of Bitcoin price fluctuations, which may have practical benefits for investors in a real market environment. The findings imply that technical market indicators outperform the characteristics produced by DAEs, and adding associated cryptocurrencies can help predict swings in the price of bitcoin. Additionally, ALEN performs 3.3% better in terms of accuracy and 3.2% better in terms of the F1 score than ALUE, which employs a uniform embedding approach.

[38] used three algorithms based on deep learning to forecast daily arrivals at an emergency room, which frequently experiences overload and seasonal increases. While the last two models are based on a newly created TFT architecture for multi-horizon time-frame forecasting, the first model is a straightforward RNN with LSTM cells. The dataset used to train the models contained a variety of pertinent variables, with the TFT models beating the LSTM model for prediction success. The hourly frequency TFT model outperforms the competition model by using layers of temporal self-attention and a smart network design to develop dependency patterns over time. The TFT model also provides greater interpretability, enabling the detection of important variables and eliminating those that provide little value. Information on what the model is attentive to regarding time-series segments can be deduced from the self-attention layer weights.

In their research, [37] addressed three issues to better understand this complex problem, which included employing advanced deep learning architectures for predicting cryptocurrency prices. The findings indicated that the chaotic and complicated nature of the market structure hinders deep learning algorithms such as LSTM and CNNs from being good predictors of the Bitcoin price. It uses a clever mechanism to detect a few hidden patterns in the random walk process that determines the path of cryptocurrency prices to achieve accurate predictions. The study concludes that additional validation techniques, complex computational methods, and ensemble methods are required to increase the precision of Bitcoin price prediction. Accurate price prediction is a difficult challenge, given the growing importance of cryptocurrencies in the financial sector; therefore, researching new methods is essential for better results.

[36] explores the difficulties and opportunities associated with academic research on financial time-series data, including different approaches to issue framing and feature extraction. Researchers aim to close the gap between academia and business by proposing a machine-learning-based forecasting approach that starts with feature extraction and selection. Hourly statistics from Solana, Bitcoin, and Ethereum were gathered from the exchange FTX and used in the study. This study investigated the problem statement of how effectively market movements can be predicted and the contribution of each feature to the forecasts for a six-hours-ahead regression job using a collection of candlestick patterns and a feature selection method. The findings demonstrate that several forecasting models provide evidence of a market's short-term predictability. When volatility was low, LSTM and ARIMA-GARCH performed the best; LSTM outperformed the other models. The experiments also highlighted the significance of feature selection for some time pertinent to the prediction window and non-stationary indicators. Finally, the data exhibit a significant mean-reverting pattern and may be roughly predicted by a Naïve walk.

## 3 Methodology

**Figure 1:**
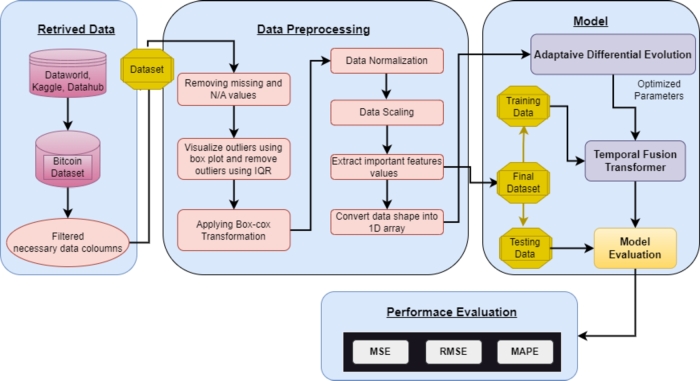
The image illustrates a workflow for Bitcoin price prediction encompassing data retrieval, preparation with cleaning and outlier detection, preprocessing with normalization and correlation analysis, model training using ADE-TFT, and performance evaluation through regression metrics.

Fig. 1 shows the complete workflow of Bitcoin price prediction by leveraging the ADE-TFT. The workflow is divided into four phases: data retrieval, preprocessing, model construction and performance evaluation. In the data retrieval phase, the daily transaction dataset for Bitcoin is gathered from many sources (Kaggle, DataHub, and DataWorld) and integrated such that it has at least eight years of daily transaction records from the past and only extracts the key elements required for this study.

Before preparing the data, an essential stage is filtering the necessary data columns, which entails choosing only the appropriate data columns needed for analysis. By doing so, the amount of data that must be processed can be decreased, enhancing the effectiveness of the analysis. Raw data must be changed and cleaned by removing N/A and missing values rather than values that can be filled with mean, median, or mode values for the respective features. The next step involved the use of a box plot library to visualize the outliers. Any data points that are noticeably distinct from the rest of the dataset can be identified using this graphical approach. Once they have been located, outliers are eliminated using the Interquartile Range (IQR) approach to ensure that they do not harm the findings of the analysis. The IQR method, which calculates the difference between a dataset's upper and lower quartiles, is frequently used to identify the range of values that fall within the middle 50% of the dataset. Any data point that deviated from the IQR range was eliminated as an outlier. After removing outliers, it is crucial to ensure that the remaining data are normally distributed. This skewed or unusual data prediction may result in inaccurate or unreliable analytical output. The Box-Cox transformation is frequently used as a well-known data preparation approach to normalize data and lessen its skewness to solve this issue. To transform the data such that it conforms to a normal distribution, this process involves raising the values of the data to power and then estimating the best lambda. The Box-Cox transformation improves the distribution of data, producing more reliable and accurate analytical results.

Further, after preparing and cleaning the data, they need to be normalized and scaled to ensure uniformity and standardization. In this study, the z-score method was used for data normalization, whereby the data were converted into a standard normal distribution with a mean of 0 and a standard deviation of 1. This helps neutralize variations owing to the different scales of various variables within the dataset. Min-max scaler was used and each data point got the same weightage. It was used to scale the data between zero and one. This scaling is particularly useful when relative differences between the values are important, but not absolute ones. In addition to normalizing or scaling, other important attributes that are highly related to the target variable must be extracted or identified from such features. Pearson's correlation was used in this study to determine the linear relationship between the two variables. It is feasible to minimize the dimensionality of the data and choose only the most important characteristics for analysis by locating the strongly associated features. Reducing the amount of noise in the data and increasing the signal-to-noise ratio can produce analytical findings that are more accurate and effective.

**Figure 2:**
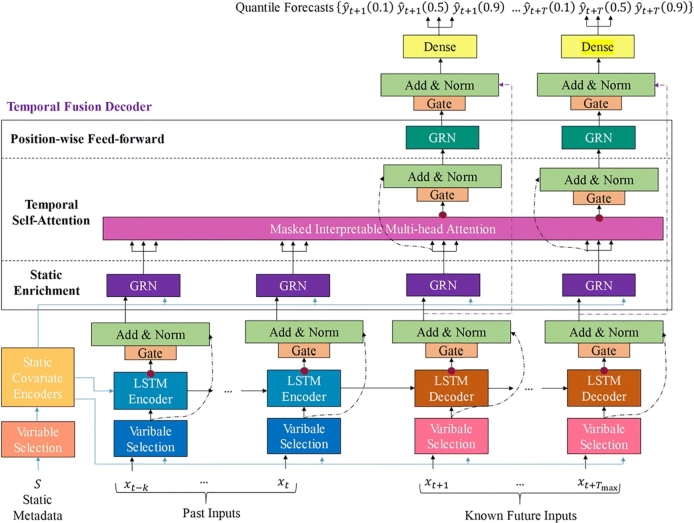
Illustrates the TFT's model framework. TFT's ability to efficiently generate feature representations for each type of input using fundamental components improves the performance of many prediction tasks. Components of TFT include an input embedding layer, Static Covariate Encoders, a Gated Residual Network, a Temporal Fusion Decoder and an output layer.

Fig. 2 shows the complete operation of the Temporal Fusion Transformer (TFT) model. TFT is a deep-learning algorithm that leverages the benefits of both transformers and temporal convolutional networks (TCNs) to predict time-series data. The TFT model is built on an attention-based deep neural network architecture to integrate multi-horizon forecasting with a clear understanding of temporal dynamics. The attention mechanism adds new levels of interpretability to the model. TFT involves complex inputs such as static covariates, known future inputs, and exogenous time series, which are only observed historically. By using several techniques, including sequence-to-sequence layers for local processing of observed inputs, sample-dependent variable selection to weed out unnecessary inputs, a static covariate encoder for encoding context vectors, and temporal self-attention that makes use of a decoder to capture long-term dependencies in the dataset, the model combines accurate forecasting with interpretability.

Other key features of the Temporal Fusion Transformer are:

a) A Gating Residual Network (GRN) supports multiple datasets and dilemmas by dropping unnecessary elements of architecture to avoid nonlinear processing. b) Variable Selection Network (VSN) utilizes a GRN under the hood for its filtering capabilities, producing a normalized vector of weights. Finally, the output is calculated using a linear combination. c) Static covariate encoders transform input variables into a fixed-size vector representation. This vector representation, sometimes referred to as a contextual feature vector, includes details of the static variables that might provide the model with extra context and boost its prediction precision. TFT may exploit extra information and increase prediction accuracy by integrating static covariates via the implementation of a static covariate encoder, especially when analyzing time-series data with complex inputs. d) Temporal processing entails the creation of temporal connections from known time-varying inputs and either short- or long-term data. For short-term local processing, features are created by utilizing the sequence-to-sequence layers of LSTM encoders and decoders for past inputs and observations. TFT multi-head attention component captures the long-term relationships between inputs and is used to capture long-term dependencies. This attention method ranks the input variables according to the magnitude of their attention weights, enabling the detection and removal of underperforming variables. TFT can successfully capture both short- and long-term temporal connections by integrating sequence-to-sequence layers with a multi-head attention block, allowing it to provide more precise predictions. e) Multi-level prediction intervals use a Gaussian mixture model to provide prediction intervals to account for the variability of the target variable. Generate prediction intervals with various degrees of confidence based on estimations of the conditional distribution at each prediction horizon, which can help make decisions in the face of uncertainty. This provides accurate and reliable forecasts.

**Figure 3:**
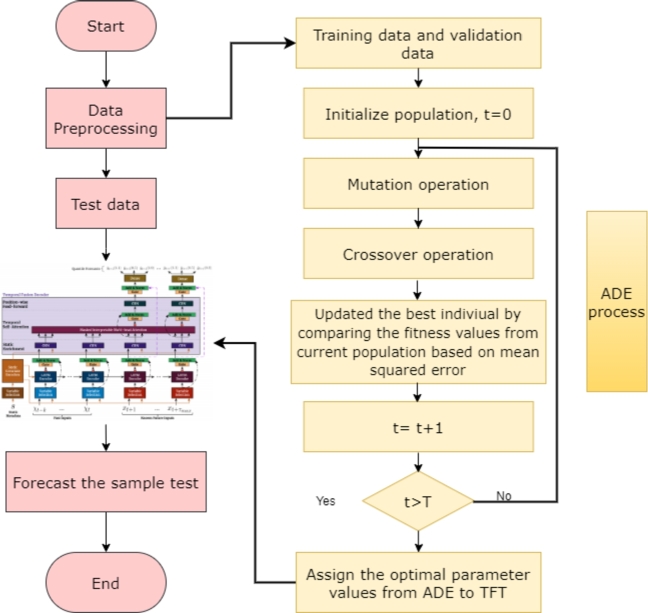
ADE process includes training and validation data, population initialization, mutation operation, crossover operation and finding optimal parameters for TFT.

The properties of the TFT model significantly impact its accuracy and performance. However, selecting the best set of parameters can be challenging. An efficient and trustworthy optimization technique is required to solve this problem. One such algorithm that has proven to be both simple and effective is adaptive differential evolution (ADE), which belongs to the class of evolutionary optimization methods. Evolutionary optimization algorithms offer the benefit of searching across a wider range of parameters, including the input step size, which is not accessible in many other tuning approaches, as compared to other neural network parameter tuning techniques. In this study, ADE was used to determine the optimal combination of hyperparameters for the TFT model, including the number of time steps, learning rates, batch sizes, hidden layer counts, consecutive hidden layer counts, and attention head count. Fig. 3 shows the ADE process that incorporates the TFT model for performing prediction tasks.

The initial setup of the ADE algorithm involved setting up its parameters and population. This involves determining the maximum number of iterations allowed (T), crossover factor range (CR), range of the mutation factor (F), size of the population (NP), and gene range to be included in the population. The range of values that each hyperparameter could have was determined using the gene range. A random population was formed based on the gene range to provide a diversified population. Step 3: Primary stage of optimization. Mutation, crossover, and selection activities are used to form the population for the upcoming generation. Equation (5) is used to calculate the mutation factor, where ‘$F_{max}$’ and ‘$F_{min}$’ stand for the variation factor's maximum and minimum values, respectively.

(5) $$F=F_{\text{min}}+(F_{\text{max}}-F_{\text{min}})\cdot \frac{1}{1+e^{10\cdot (0.5-\frac{t}{T})}}$$

The dataset was divided into three parts: the training set, validation set, and testing set. 70% of the data was reserved for the training set, while 15% was allocated to both the validation and testing sets. The validation set Mean Absolute Percentage Error (MAPE) was used to compute the fitness value. The values ‘T’ and ‘t’ stand for the maximum and current iteration counts, respectively. The third step is repeated a predetermined number of times to enable the ADE algorithm to converge to the ideal hyperparameters. The variable ‘t’ is for a specific point in time within the sequence i.e., it could represent the price on a particular day. On the other hand, ‘T’ identifies the complete length of the time series or the maximum time taken for the analysis. The ADE individual with the highest fitness value was selected to obtain the best hyperparameters, which were then assigned to the TFT model. Subsequently, by utilizing both the training and validation datasets, the TFT model was trained. This step ensures that the TFT algorithm can accurately identify and interpret key characteristics of the data and produce accurate forecasts. Predictions on the test dataset were performed using the TFT model with the best training performance. This ensures that the TFT algorithm can produce accurate and reliable forecasts using fresh untested data. Algorithm 1 shows the detailed steps performed by the ADE algorithm for optimal feature and parameter selection.

**Algorithm 1:**
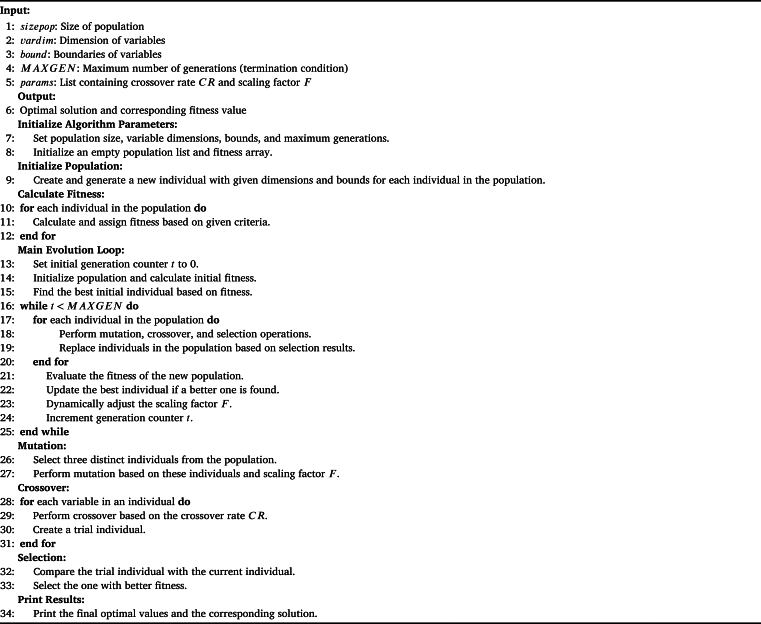
Adaptive Differential Evolution.

Algorithm 2 shows the step carried out by TFT to predict bitcoin prices.

**Algorithm 2:**
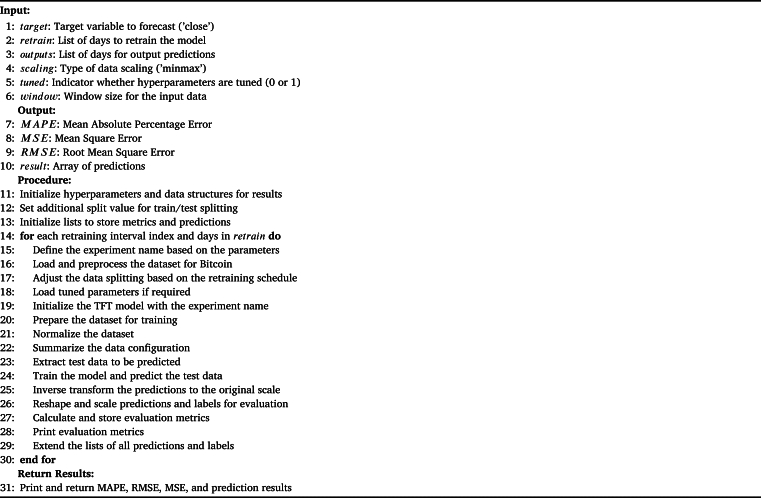
Forecasting bitcoin prices using TFT.

### 3.1 Mathematical formulation of ADE-TFT model

The ADE-TFT model joins numerous advanced methods to manage the temporary dependencies and complicated patterns in time-series data. Below, we provide the mathematical definition and explanation of the basic components of the ADE-TFT model. The input data to the ADE-TFT model consists of two parts: past observation and future data.

#### 3.1.1 Input embeddings

Let $X_{\text{past}}=\{x_{t-n},x_{t-n+1},\ldots ,x_{t}\}$ and $X_{\text{future}}=\{x_{t+1},x_{t+2},\ldots ,x_{t+m}\}$ be the past and future input sequences, respectively.

The embedding for the past and future inputs is represented as:

$$E_{\text{past}}=\text{Embedding}(X_{\text{past}})$$

$$E_{\text{future}}=\text{Embedding}(X_{\text{future}})$$

#### 3.1.2 Temporal attention mechanism

Next, the Temporal Fusion Transformer employs a multi-head attention procedure to catch temporal dependencies.

$$\alpha_{i}=\text{softmax}\left(\frac{(QW_{i}^{Q}){\left(KW_{i}^{K}\right)}^{⊤}}{\sqrt{d_{k}}}\right)$$

where $Q=E_{\text{past}}$, $K=E_{\text{past}}$, and $V=E_{\text{past}}$ are the query, key, and value matrices, respectively, and $W_{i}^{Q}$, $W_{i}^{K}$, and $W_{i}^{V}$ are the learned weight matrices for the *i*-th head.

The outputs of all heads are concatenated and linearly transformed:

$$Z=\text{Concat}(\alpha_{1}VW_{1}^{V},\alpha_{2}VW_{2}^{V},\ldots ,\alpha_{h}VW_{h}^{V})W^{O}$$

#### 3.1.3 Static covariate encoders

Static covariate features are those features that do not change over time such as certain demographic features and geographical location. For the ADE-TFT model, static covariate features are encoded to represent the temporal behavior of the model. The static encoder manages these static covariates and combines them into the model to have an impact on the temporal attention and prediction methods.

$$E_{\text{static}}=\text{StaticEncoder}(S)$$

The encoded static covariates, $E_{\text{static}}$, are then combined into the model to influence the attention mechanism and prediction layers.

#### 3.1.4 Gated residual network

To further enhance the learning experience and capacity, the ADE-TFT model employs a critical component called Gated Residual Network (GRN). To effectively propagate the gradients GRN uses residual connections and gating mechanisms.

$$H=\text{ReLU}(W_{1}X+b_{1})⊙\sigma (W_{2}X+b_{2})+X$$

where:

• **X** is the input to the GRN.
• $W_{1}$ and $W_{2}$ are learned weight matrices.
• $b_{1}$ and $b_{2}$ are bias vectors.
• ReLU is the Rectified Linear Unit activation function.
• *σ* is the sigmoid activation function.
• ⊙ denotes element-wise multiplication.

The GRN output, **H**, combines the transformed input through a ReLU activation and a gated mechanism using a sigmoid function. The residual connection **X** allows the original input to be added back to the transformed input, enhancing gradient flow and improving learning dynamics.

#### 3.1.5 Decoder

The decoder combines the encoded interpretations from the temporal attention mechanism, static covariates, and the Gated Residual Network (GRN) to produce the final output.

$${\overset{ˆ}{y}}_{t}=\text{Decoder}(Z,E_{\text{future}},E_{\text{static}})$$

where:

• **Z** is the output from the temporal attention mechanism, representing the combined past embeddings.
• $E_{\text{future}}$ is the embedding of future known inputs.
• $E_{\text{static}}$ is the embedding of static covariates.
• Decoder is a function that integrates these embeddings to produce the final prediction ${\overset{ˆ}{y}}_{t}$.

The decoder processes these inputs to predict the target feature at each time step *t*. The combination of temporal attention outputs, future embeddings, and static covariates enables the model to make accurate and context-aware predictions.

#### 3.1.6 ADE-TFT loss function

Finally, the loss function in the ADE-TFT model finds the difference between the predicted values and the actual values, typically based on error metrics such as Mean Absolute Percentage Error (MAPE), Mean Squared Error (MSE), and Root Mean Square Error (RMSE). The loss function further guides the ADE-TFT training process by feeding a gradient to adjust the model parameters.

The total loss function L can be a combination of these error metrics to balance their effects:

L=α⋅MAPE+β⋅MSE+γ⋅RMSE

where *α*, *β*, and *γ* are the weights assigned to each error metric to balance their contributions to the total loss.

### 3.2 Challenges

Several challenges were encountered while predicting cryptocurrency market movements within the unstable global financial markets. First, it was noticed that the dataset contains the intrinsic volatility of cryptocurrencies due to rapid price fluctuations which complicated the development of an accurate prediction model. It was also noticed that the cryptocurrency market is sensitive to regulatory fluctuations, geopolitical events, and economic trends. Therefore, these factors introduce uncertainty and noise into prediction models. Moreover, market mawkishness, often prompted by social media and news outlets, plays an important role in price changes, forcing clever sentiment analysis tools to portray this dynamic. Finally, the distributed and comparatively undeveloped nature of the cryptocurrency market leads to broken data sources and varying reporting standards, making it challenging to obtain clean and consistent datasets for consideration.

### 3.3 Regression metrics

Statistical measures that indicate how well a model can predict continuous data, such as Mean Squared Error (MSE), Root Mean Squared Error (RMSE), and Mean Absolute Percentage Error (MAPE), are used to evaluate a model's performance for regression problems [11]. These measurements are used to determine the precision and accuracy of the model's predictions, and the extent of the unpredictability of the outcome variable can be explained by the independent variables. The utilization of these metrics includes training the regression model, making predictions, and determining MSE, RMSE, and MAPE to measure errors. The best regression fitting model can be selected based on lower values of these metrics.

Mean Squared Error (MSE): It counts the average of the squared anomalies gap among the forecasted outcome and ground truth results.

(6) $$MSE=\frac{1}{n}\sum_{i=1}^{n}{\left(actual_{i}-predicted_{i}\right)}^{2}$$

where *n* is the number of instances in the dataset, $actual_{i}$ is the ground truth values of the objective variable for observation *i*, and $predicted_{i}$ is the predicted value of the target variable for observation *i*. Moreover, MSE can be used for hyperparameter tuning, regularization, model cross-validation, bias-variance tradeoff, error analysis and outlier detection.

Root Mean Squared Error (RMSE): It quantifies the square root average of the squared anomaly gap between the forecasted outcome and the ground truth results. It was calculated as the square root of the MSE.

(7) $$RMSE=\sqrt{\frac{1}{n}\sum_{i=1}^{n}{\left(actual_{i}-predicted_{i}\right)}^{2}}$$

where *n* is the number of instances in the dataset, $actual_{i}$ is the ground truth values of the objective variable for observation *i*, and $predicted_{i}$ is the predicted value of the target variable for observation *i*. A low RMSE value indicates that the predicted values are close to the actual values indicating the model's good fit.

Mean Absolute Percentage Error (MAPE): It assesses the average percentage deviation between the forecasted outcome and the ground truth results. It quantified and expressed the proportional difference between the expected and actual values.

(8) $$\text{MAPE}=\frac{1}{n}\sum_{i=1}^{n}\left|\frac{{\text{Actual Value}}_{i}-{\text{Predicted Value}}_{i}}{{\text{Actual Value}}_{i}}\right|\times 100$$

where *n* is the number of instances in the dataset, $actual_{i}$ is the ground truth values of the objective variable for observation *i*, and $predicted_{i}$ is the predicted value of the target variable for observation *i*. A low MAPE value suggests that the trained model prediction is close to the actual Bitcoin prediction, in terms of prediction, indicating acceptable model performance. Moreover, cross-validation can also ensure that the trained model is generic and does not suffer from overfitting.

### 3.4 Incorporating sentiment analysis into ADE-TFT

Various NLP libraries and tools such as NLTK and SpaCy were used to perform sentiment analysis and incorporate it into the ADE-TFT model. First, the text preprocessing step was performed, separating the text into tokens. Next, the common words that do not contribute to sentiment such as and, is, and the were filtered out. Next, through the process of Lemmatization, the words were reduced to their root form. Using techniques such as polarity scores and aggregating scores, first numerical values were assigned to the sentiment to represent negative, positive and neutral sentiment and then sentiment scores were summarized for a certain period to create time series sentiment data. Next, the aggregated sentiments score was transformed into a form that can be provided to the ADE-TFT model. Next, time series alignment was performed where sentiment attributes were aligned with the corresponding time frame of other input attributes such as volume and price data. Next, sentiment attributes were combined with input attributes to form a complete and comprehensive feature set. Next, TFT was used to assign various weights to sentiment attributes at different time stamps. This allows the ADE-TFT model to focus more on meaningful sentiment alteration with greater market influence. Furthermore, the ADE-TFT model was trained in sentiment attributes and historical market data. Lastly, metrics such as RMSE, MAPE, and MSE were used to evaluate the ADE-TFT model performance.

This study demonstrated that various causes beyond historic price data, market sentiment, and social media trends prompt cryptocurrency markets. Sentiment analysis, which includes parsing and decoding the feelings and thoughts communicated in social media posts and news articles offers a valued understanding of the market's state. By integrating these perceptions, the prediction model can better foresee market changes that are steered by investor sentiment and peripheral events. Furthermore, the study highlights the essential for sophisticated normalization and data preprocessing techniques to process the unpredictability and randomness of cryptocurrency data. Advanced AI techniques are critical for efficiently normalizing and preprocessing this data, guaranteeing that the model is trained on reliable and appropriate information.

## 4 Experiments and results

The TFT model was trained using historical Bitcoin data from 17-09-2014 to 25-11-2022, which includes 4857377 records with several attributes.

**Figure 4:**
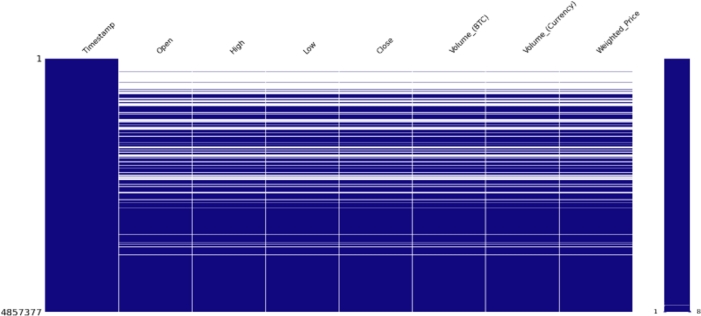
Missing values.

**Figure 5:**
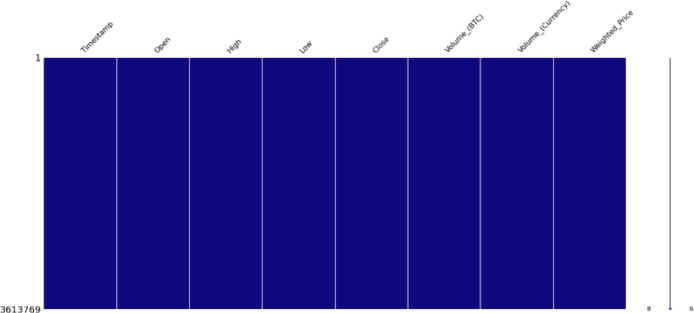
After removing missing values.

The dataset used for this research shown in the following Fig. 4 has many entries with missing values. Missing values were around 25% which were removed, and the resulting dataset is shown in Fig. 5.

**Figure 6:**
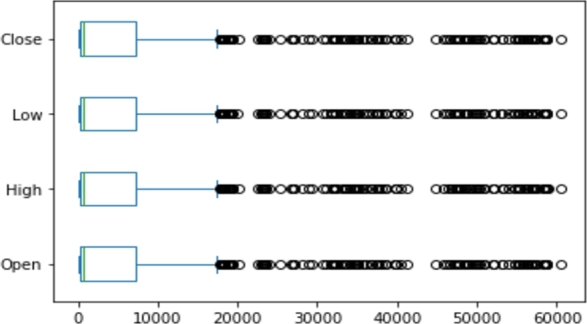
Outliers in dataset.

This research study aims to estimate the $X_{\tau_{+}1}$ to $X_{\tau_{m}ax}$ price of bitcoin using the currently available data. Therefore, the dataset was organized according to date and time feature, and for this $Time_{i}dx$, Month, and Year columns were derived from the Timestamp. Fig. 6 shows a box plot to visualize the scattered and skewed data to solve the dataset's outliers issue. The data was changed into a normal distribution where μ=0 and σ=1, by using box-cox transformation and distribution techniques.

**Figure 7:**
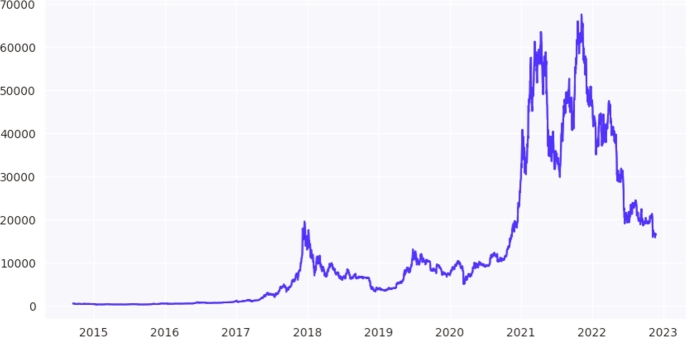
Trend of weighted price feature over the years.

To forecast the Weighted_Price feature, we must evaluate its trends and patterns over time. For this, the Weighted_Price graph is plotted in Fig. 7 to observe the behavior and seasonality of the price of Bitcoin in prior years. The Bitcoin price in $USD was almost stable from 2014-2016 to 2017 it gradually increased from after to 2017-2021. Data were divided into three sets. The training data covered the period from 2014:09 to 2019:12, which is dependent on a 63-month training batch; the validation data covered the period from 2020:01 to 2022:02 with 26 months, and the testing data covered the period from 2022:02 to 2022:11 with 09 months of observations.

### 4.1 Hyperparameters selection

**Figure 8:**
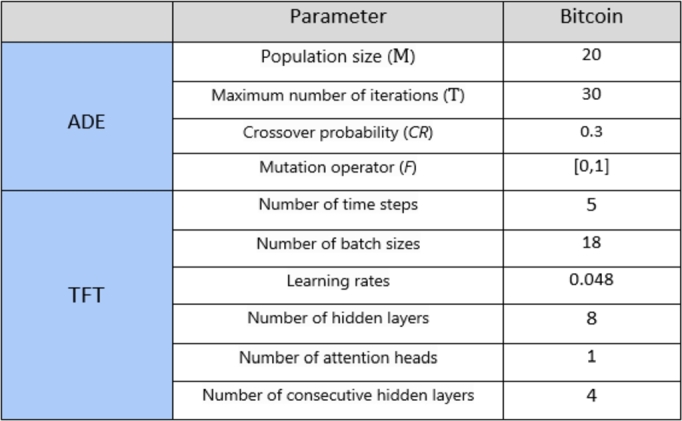
Hyperparameters of ADE-TFT model.

The range for TFT model hyperparameters is selected between: The range for batch sizes[5,20]; time steps[2,12]; attention heads[1,4]; hidden layers[2,4,8]; and successive hidden layers[2,4,8]. While keeping all other hyperparameters constant, it is shown in below Fig. 8. Tests on the TFT model were conducted using the hidden layer sizes 2, 4, and 8. In addition, 100 epochs were specified with patience of 15 before the training process was terminated if the model was not converging.

### 4.2 Performance evaluation

**Figure 9:**
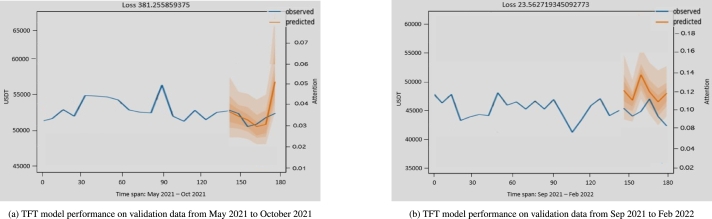
TFT model performance on validation data on different dates.

The best model weights were saved after the model was trained on the training dataset. The graphs in Figs. 9a and 9b show some of the prediction quantiles from the validation dataset. Forecasts appeared accurate, and the prediction samples were good enough as the model learned and converged on different points of the time index as fluctuations occurred. The grey lines represent how much weight the model gives to certain periods in time when generating the forecast.

**Figure 10:**
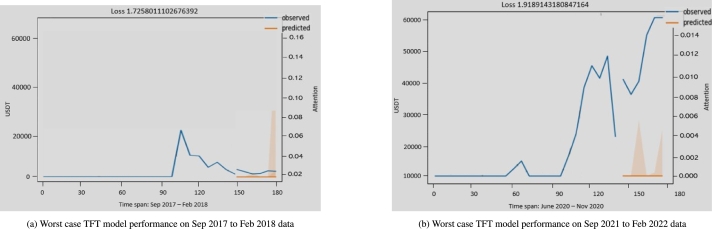
TFT model worst-case performance on different dates.

Figs. 10a, 10b show the TFT model learning pattern and its poor convergence, making it an unreliable forecasting model. The reason for poor convergence could be that Bitcoin prices are affected by events such as politics, the supply chain of money, money laundering, the compactness of other currencies, and people's sentiments, which make the TFT model prediction unreliable.

### 4.3 TFT model prediction on testing data

**Figure 11:**
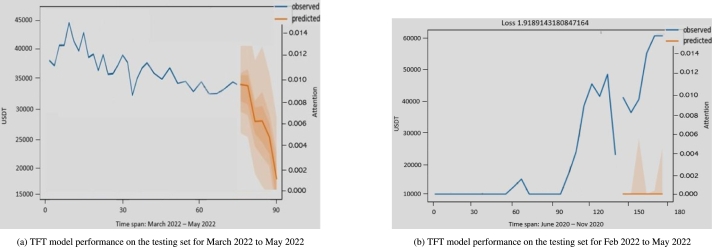
TFT model performance on testing set.

Because the dataset contained covariates and the encoder length was set to the previous 24 months of data when predicting the price, the decoder data were created using forward filing by repeating the covariate features from the most recent known points. The encoder and decoder were integrated to predict the unseen data used as the testing set. The graphs shown in Fig. 11a and Fig. 11b are a few examples of random sample graphs that forecast future prices over different periods.

**Figure 12:**
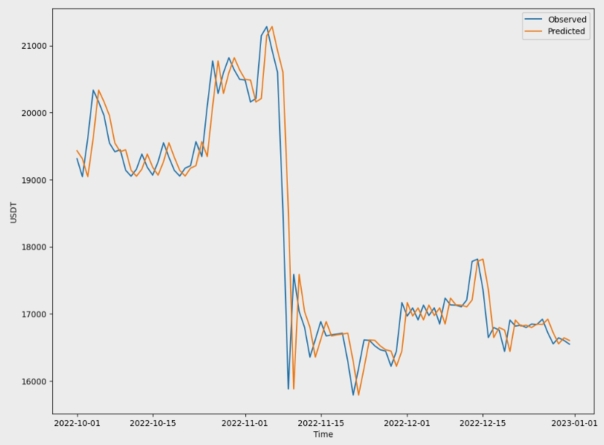
The performance of a predictive model for USDT prices over time.

The performance of the predictive model for USDT prices over time is shown in Fig. 12. The blue line represents the historical prices used to train the model. Next, the correctness of the model was verified using orange unseen data. This alignment suggests a model that is capable of accurately predicting USDT prices, which is crucial for making investment decisions.

**Figure 13:**
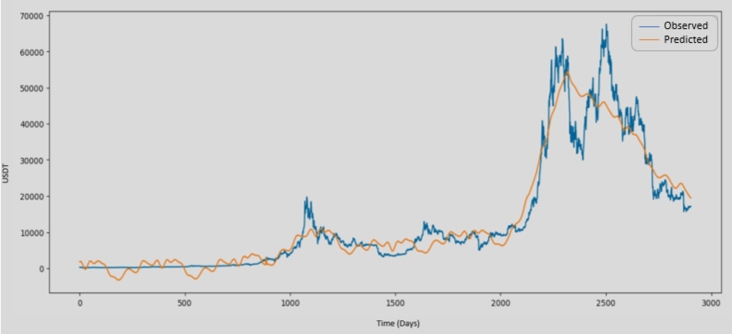
Comparing model predictions with actual points.

Fig. 13 shows how well the trained model predicts the data point and coverage from time to time when compared to the actual values of Bitcoin historical data.

Different hidden layers [2,4,8] of TFT model versions were examined, as indicated in Table 1; however, the 8th hidden layer outperformed the others when combined with additional hyperparameters.

**Table 1:** Results comparison between the proposed system and the existing models.

Citation	Existing Models	Study Currency	RMSE	MAPE
	Autoregressive integrated moving average (ARIMA),		302.53	42.239
[4]	Long Short-Term Memory (LSTM)	BTC-USDT	603.68	87.413
	Gated Recurrent Unit (GRU)		381.34	49.808

[48]	Least Squares Support Vector Machine (LSSVM)	BTC-USD	272.155	37.720
	Neural Networks with Backpropagation		540.087	74.935

Proposed	ADE-TFT(h=2)		**209.74**	**31.0104**
model	ADE-TFT(h=4)	BTC-USD	**178.68**	**29.3295**
	ADE-TFT(h=8)		**167.12**	**23.1734**

**Figure 14:**
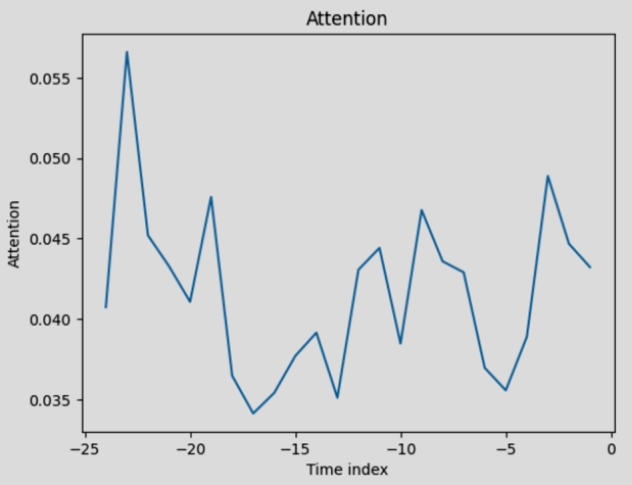
Attention to various latency orders.

Fig. 14 demonstrate the ADE-TFT model's interpretable results. The explanation is divided into three parts: the relevance of various lag orders, previous inputs' importance, and future variables' importance.

**Figure 15:**
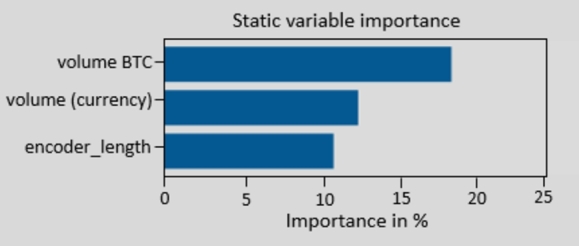
Importance of static variables.

The model identifies patterns and correlations between the input characteristics (encoder variables) and the target variable (decoder variable) using static variables that provide constant information. Fig. 15 shows the importance of variables that are constant throughout the testing phase.

The TFT model results for the price prediction of cryptocurrencies show that more accuracy can be obtained by using the pre-trained model or by training on different hidden layer sizes with different combinations of hyperparameters with more resource consumption. In addition to these deficiencies, the model learns many patterns that converge with more data points and precisely predicts the quantile values.

The ADE-TFT model utilizes deep learning, specifically transformer architectures, to obtain long-term dependencies and dense temporal arrangements in the data, which traditional models rarely achieve. This facilitates the ADE-TFT to successfully understand historical price data and recognize subtle, non-linear relationships that manipulate Bitcoin prices. Secondly, the ADE-TFT model combines multiple data streams, including geopolitical events, market sentiment, and transactional relationships, allowing for a more widespread analysis of the features shaping Bitcoin values. This multi-source data integration improves the model's power to learn relevant signals from the noise, resulting in more robust predictions. Furthermore, the ADE-TFT model uses an advanced hidden layer configuration (h=8), which drastically improves its capability to model sophisticated patterns and connections within the data. This depth of representation is put in to decrease prediction errors, as demonstrated by reduced MAPE, MSE, and RMSE values compared to traditional models. Lastly, the model's architecture incorporates advanced normalization techniques that improve its ability to control the irregularities and volatility present in cryptocurrency markets. By utilizing these methods, the ADE-TFT model can adjust to moving market conditions more efficiently than traditional approaches.

## 5 Conclusion, limitations and future work

Transformer models have been among the most extensively investigated topics. These models are implemented in multiple domains, mostly in time-series prediction or in large-language models. This study presented a complete procedure, from data collection and data cleaning to model training and evaluation. Based on the results in Table 1 the TFT with h=8 performs slightly better than the TFT with a hidden layer size 2,4 while setting the other parameters the same. TFT h=8 excels in producing more precise probabilistic ranges.

This study uses a Temporal Fusion Transformer (TFT) to predict Bitcoin prices. Nonetheless, this study has the following limitations.

1. **Model Complexity and Training Time:** The complexity of the Temporal Fusion Transformer (TFT) model significantly increases the amount of computer resources required for training, particularly when working with a large number of hidden layers. This inhibits the ability of the model to be retrained in response to fresh data and prolongs the development cycle.
2. **Data Quality and Availability:** Although this study has made an effort to include a variety of data points, the model's predictions may be greatly impacted by the quantity and quality of external data, such as news on regulations or market sentiment. The model's ability to completely understand market dynamics may have been restricted by a lack of access to trustworthy and up-to-date data sources.
3. **Generalization Capability:** It was not determined whether the model could be applied to other cryptocurrencies or financial markets. The applicability of the model outside the parameters of this study may be limited by the peculiarities and instability trends associated with Bitcoin, which may not be typical of other assets.

Several improvements could be made to the performance of the ADE-TFT model. Adding additional elements to the dataset, such as market sentiment or regulatory news, can impact cryptocurrency prices. By preprocessing and adding this new data throughout the training procedure, the TFT algorithm can obtain a more comprehensive understanding of market trends. The next enhancement is the possibility that the performance of the TFT model may be affected by the normalization method used. While the standard method for data transformation uses standard z-score scalars, experiments with different normalization techniques may produce superior results. For instance, one may consider employing robust scalars to reduce the impact of outliers in the data on Bitcoin pricing. The model can produce forecasts that are more accurate by reducing the influence of extreme values.

The practical implications of the study include enhanced prediction accuracy, timely insights, informed decision-making, investment portfolio optimization, market volatility and downturn predictions, market analysis, and developing Sentiment-Driven Strategies.

The future work includes the ADE-TFT model expansion to the other Cryptocurrencies, cross-market validation, incorporation of additional features related to cryptocurrencies, advanced sentiment analysis (which includes real-time social media streams and multi-lingual sentiment analysis), real-time predictions, model optimization and efficient resource management. Moreover, explainable AI can be integrated with ADE-TFT to make it understandable to the common person.

Despite being a fascinating subject, it has been established that predicting the spread between a cryptocurrency and its futures contracts is challenging because of the high volatility and huge amount of noise the time series includes. As a result, this time series requires novel approaches for integrating the initial processing and forecasting algorithms.

## CRediT authorship contribution statement

**Arslan Farooq:** Writing – review & editing, Writing – original draft, Visualization, Methodology, Data curation, Conceptualization. **M. Irfan Uddin:** Methodology, Formal analysis, Data curation, Conceptualization. **Muhammad Adnan:** Methodology, Formal analysis, Conceptualization. **Ala Abdulsalam Alarood:** Funding acquisition, Formal analysis. **Eesa Alsolami:** Writing – review & editing, Project administration, Investigation, Funding acquisition. **Safa Habibullah:** Writing – review & editing, Project administration, Funding acquisition.

## Declaration of Competing Interest

The authors declare that they have no known competing financial interests or personal relationships that could have appeared to influence the work reported in this paper.

## Data Availability

Data will be made available on request

## References

[1] Adhikari Ratnadip, Agrawal Ramesh K. (2013). An introductory study on time series modeling and forecasting. https://arxiv.org/abs/1302.6613.

[2] Akyildirim Erdinc, Corbet Shaen, Katsiampa Paraskevi, Kellard Neil, Sensoy Ahmet (2020). The development of bitcoin futures: exploring the interactions between cryptocurrency derivatives. Finance Res. Lett..

[3] Amin Samina, Uddin M. Irfan, Al-Baity Heyam H., Zeb M. Ali (2021). Computers, Materials & Continua.

[4] Awoke Temesgen, Rout Minakhi, Mohanty Lipika, Satapathy Suresh Chandra (2020). Communication Software and Networks: Proceedings of INDIA 2019.

[5] Barba Maggi Lida (2017). III Concurso Latinoamericano de Tesis de Doctorado (CLTD-CLEI)-JAIIO 46 (Córdoba, 2017).

[6] Bouteska Ahmed, Abedin Mohammad Cocco, Hajek Petr, Yuan Kunpeng (2024). Cryptocurrency price forecasting–a comparative analysis of ensemble learning and deep learning methods. Int. Rev. Financ. Anal..

[7] Amin Samina, Uddin M. Irfan, Al-Saeed Duaa H., Khan Atif, Adnan Muhammad (2021). Complexity.

[8] Box George E.P., Jenkins Gwilym M., Reinsel Gregory C., Ljung Greta M. (2008).

[9] Breedon Francis, Ranaldo Angelo (2013). Intraday patterns in fx returns and order flow. J. Money Credit Bank..

[10] Caldas Francisco M., Soares Cláudia (2022). A temporal fusion transformer for long-term explainable prediction of emergency department overcrowding. https://arxiv.org/abs/2207.00610.

[11] Chicco Davide, Warrens Matthijs J., Jurman Giuseppe (2021). The coefficient of determination r-squared is more informative than smape, mae, mape, mse and rmse in regression analysis evaluation. PeerJ Comput. Sci..

[12] de Azevedo Takara Lucas, Alves Portela Santos André, Mariani Viviana Cocco, dos Santos Coelho Leandro (2024). Deep reinforcement learning applied to a sparse-reward trading environment with intraday data. Expert Syst. Appl..

[13] Deka Ganesh Chandra, Kaiwartya Omprakash, Vashisth Pooja, Rathee Priyanka (2018). Applications of Computing and Communication Technologies: First International Conference, ICACCT 2018, Delhi, India, March 9, 2018, Revised Selected Papers, vol. 899.

[14] Derbentsev V., Babenko V., Khrustalev Kirill, Obruch Hanna, Khrustalova Sofiia (2021). Comparative performance of machine learning ensemble algorithms for forecasting cryptocurrency prices. Int. J. Eng..

[15] Di Lorenzo Renato (2013).

[16] Dudek Grzegorz, Fiszeder Piotr, Kobus Paweł, Orzeszko Witold (2024). Forecasting cryptocurrencies volatility using statistical and machine learning methods: a comparative study. Appl. Soft Comput..

[17] Fang Sheng, Cao Guangxi, Egan Paul (2023). Forecasting and backtesting systemic risk in the cryptocurrency market. Finance Res. Lett..

[18] Fassas Athanasios P., Papadamou Stephanos, Koulis Alexandros (2020). Price discovery in bitcoin futures. Res. Int. Bus. Finance.

[19] Feng Guoce, Zhang Lei, Ai Feifan, Zhang Yirui, Hou Yupeng (2022). An improved temporal fusion transformers model for predicting supply air temperature in high-speed railway carriages. Entropy.

[20] Hewage Pradeep, Behera Ardhendu, Trovati Marcello, Pereira Ella, Ghahremani Morteza, Palmieri Francesco, Liu Yonghuai (2020). Temporal convolutional neural (tcn) network for an effective weather forecasting using time-series data from the local weather station. Soft Comput..

[21] Hotait Hassane, Chiementin Xavier, Rasolofondraibe Lanto (2021). Intelligent online monitoring of rolling bearing: diagnosis and prognosis. Entropy.

[22] Hyndman R.J., Athanasopoulos G. (2021).

[23] Jeet Param, Vats Prashant (2017).

[24] Jiang Qiang, Tang Chenglin, Chen Chen, Wang Xin, Huang Qing (2019). Proceedings of the Twelfth International Conference on Management Science and Engineering Management.

[25] Kumar Deepak, Rath S.K. (2020). Artificial Intelligence and Evolutionary Computations in Engineering Systems.

[26] Lai Kin Keung, Yu Lean, Wang Shouyang, Huang Wei (2006). Computational Science–ICCS 2006: 6th International Conference, Reading, UK, May 28-31, 2006, Proceedings, Part IV 6.

[27] Li Shiyang, Jin Xiaoyong, Xuan Yao, Zhou Xiyou, Chen Wenhu, Wang Yu-Xiang, Yan Xifeng (2019). Enhancing the locality and breaking the memory bottleneck of transformer on time series forecasting. Adv. Neural Inf. Process. Syst..

[28] Li Yang, Zheng Zibin, Dai Hong-Ning (2020). Enhancing bitcoin price fluctuation prediction using attentive lstm and embedding network. Appl. Sci..

[29] Li Zhixi, Tam Vincent (2017). 2017 IEEE Symposium Series on Computational Intelligence (SSCI).

[30] Livieris Ioannis E., Pintelas Emmanuel, Stavroyiannis Stavros, Pintelas Panagiotis (2020). Ensemble deep learning models for forecasting cryptocurrency time-series. Algorithms.

[31] López Santos Miguel, García-Santiago Xela, Echevarría Camarero Fernando, Blázquez Gil Gonzalo, Carrasco Ortega Pablo (2023). Application of temporal fusion transformer for day-ahead pv power forecasting. Energies.

[32] Mahdi Esam, Leiva Víctor, Mara'Beh Saed, Martin-Barreiro Carlos (2021). A new approach to predicting cryptocurrency returns based on the gold prices with support vector machines during the covid-19 pandemic using sensor-related data. Sensors.

[33] Meijer David (2020).

[34] Mishal Mehedi Hasan, Rakhi Nura Jannat, Rashid Fahmida, Hamid Kawsar, Morol Md Kishor, Jubair Abdullah Al, Nandi Dip (2022). 2022 25th International Conference on Computer and Information Technology (ICCIT).

[35] Muzaffar Shahzad, Afshari Afshin (2019). Short-term load forecasts using lstm networks. Energy Proc..

[36] Persson Erik (2022).

[37] Pintelas Emmanuel, Livieris I.E., Stavroyiannis Stavros, Kotsilieris Theodore, Pintelas P. (2020).

[38] Pulkkinen Eetu (2020).

[39] Reddy Lekkala Sreekanth, Sriramya P. (2020). A research on bitcoin price prediction using machine learning algorithms. Int. J. Sci. Technol. Res..

[40] Reinsel Gregory C. (2003).

[41] Ribeiro Gabriel Trierweiler, Santos André Alves Portela, Mariani Viviana Cocco, dos Santos Coelho Leandro (2021). Novel hybrid model based on echo state neural network applied to the prediction of stock price return volatility. Expert Syst. Appl..

[42] Valipour Mohammad, Banihabib Mohammad Ebrahim, Behbahani Seyyed Mahmood Reza (2012). Parameters estimate of autoregressive moving average and autoregressive integrated moving average models and compare their ability for inflow forecasting. J. Math. Stat..

[43] Warmke Craig (2024). What is bitcoin. Inquiry.

[44] Williams Billy M. (2001). Multivariate vehicular traffic flow prediction: evaluation of arimax modeling. Transp. Res. Rec..

[45] Wu Binrong, Wang Lin, Zeng Yu-Rong (2023). Interpretable wind speed prediction with multivariate time series and temporal fusion transformers. Energy.

[46] Yang Ye, Lu Jiangang (2022). A fusion transformer for multivariable time series forecasting: the mooney viscosity prediction case. Entropy.

[47] Yli-Huumo Jesse, Ko Deokyoon, Choi Sujin, Park Sooyong, Smolander Kari (2016). Where is current research on blockchain technology?—a systematic review. PLoS ONE.

[48] Zhang Shengao, Li Mengze, Yan Chunxiao (2022). The empirical analysis of bitcoin price prediction based on deep learning integration method. Comput. Intell. Neurosci..

[49] Zhao Huali, Crane Martin, Bezbradica Marija (2022).

